# Oncocytoma-Like Renal Tumor With Transformation Toward High-Grade Oncocytic Carcinoma: A Unique Case With Morphologic, Immunohistochemical, and Genomic Characterization

**DOI:** 10.1097/MD.0000000000000081

**Published:** 2014-09-26

**Authors:** Sahussapont J. Sirintrapun, Kim R. Geisinger, Adela Cimic, Anthony Snow, Jill Hagenkord, Federico Monzon, Benjamin L. Legendre, Anatole Ghazalpour, Ryan P. Bender, Zoran Gatalica

**Affiliations:** Department of Pathology, Memorial Sloan Kettering Cancer Center, New York, NY (SJS); University of Mississippi Medical Center, Jackson, MS (KRG); Department of Pathology, Wake Forest Baptist Health, Winston-Salem, NC (AC, AS); 23andMe, Mountain View (JH); Invitae, San Francisco (FM), CA; Transgenomic (BLL); Creighton University School of Medicine (ZG), Omaha, NE; and Caris Life Sciences, Phoenix, AZ (AG, RPB, ZG).

## Abstract

Renal oncocytoma is a benign tumor with characteristic histologic findings. We describe an oncocytoma-like renal tumor with progression to high-grade oncocytic carcinoma and metastasis.

A 74-year-old man with no family history of cancer presented with hematuria. Computed tomography showed an 11 cm heterogeneous multilobulated mass in the right kidney lower pole, enlarged aortocaval lymph nodes, and multiple lung nodules. In the nephrectomy specimen, approximately one third of the renal tumor histologically showed regions classic for benign oncocytoma transitioning to regions of high-grade carcinoma without sharp demarcation.

With extensive genomic investigation using single nucleotide polymorphism-based array virtual karyotyping, multiregion sequencing, and expression array analysis, we were able to show a common lineage between the benign oncocytoma and high-grade oncocytic carcinoma regions in the tumor. We were also able to show karyotypic differences underlying this progression. The benign oncocytoma showed no chromosomal aberrations, whereas the high-grade oncocytic carcinoma showed loss of the 17p region housing FLCN (folliculin [Birt–Hogg–Dubé protein]), loss of 8p, and gain of 8q. Gene expression patterns supported dysregulation and activation of phosphoinositide 3-kinase (PI3K)/v-akt murine thymoma viral oncogene homolog (Akt), mitogen-activated protein kinase (MAPK)/extracellular-signal-regulated kinase (ERK), and mechanistic target of rapamycin (serine/threonine kinase) (mTOR) pathways in the high-grade oncocytic carcinoma regions. This was partly attributable to FLCN underexpression but further accentuated by overexpression of numerous genes on 8q. In the high-grade oncocytic carcinoma region, vascular endothelial growth factor A along with metalloproteinases matrix metallopeptidase 9 and matrix metallopeptidase 12 were overexpressed, facilitating angiogenesis and invasiveness.

Genetic molecular testing provided evidence for the development of an aggressive oncocytic carcinoma from an oncocytoma, leading to aggressive targeted treatment but eventual death 39 months after the diagnosis.

## INTRODUCTION

Renal oncocytoma is considered a benign tumor. Microscopically, renal oncocytoma shows nested architecture and tumor cells with granular cytoplasm and round nuclei. The definitive diagnosis of renal oncocytoma requires adequate sampling on kidney resection specimens to exclude regions of solid architecture, nuclear irregularity, and perinuclear cytoplasmic clearing. Such features are seen in eosinophilic chromophobe carcinoma, a malignant tumor that can mimic renal oncocytoma. We present an oncocytoma with transformation to a high-grade oncocytic carcinoma, supported by extensive genomic analysis.

## CASE REPORT/CLINICAL HISTORY

The patient is a 74-year-old man with no family history of cancer who presented with hematuria. Computed tomography (CT) showed an 11 cm heterogeneous multilobulated mass in the right kidney lower pole, enlarged aortocaval lymph nodes, and multiple lung nodules. A CT-guided fine needle aspirate (FNA) of an enlarged aortocaval lymph node showed large oncocytic cells with high-grade cytology, which was diagnosed as high-grade carcinoma. A radical nephrectomy with retroperitoneal lymph node dissection was then performed. Subsequently, the patient was placed on Sunitinib that controlled his metastatic disease. After 16 months post operation, the patient was changed to Axitinib and at 20 months post operation, Temsirolimus was added. Although never recurrence free, the patient nearly lived for an additional 20 months; eventually succumbing to his metastatic disease 39 months post operation.

## METHODS

### Genomic Analysis

#### Virtual Karyotype With SNP-Based Array Analysis

Tumor enrichment was enabled by selecting only tissue blocks with pure histology. One tissue block contained pure high-grade oncocytic carcinoma with 2 tissue blocks with pure benign oncocytoma-like regions. A matched normal tissue block for germline controls was obtained from adjacent pure normal kidney tissue. After tumor enrichment via manual microdissection, DNA was obtained from 10 µm paraffin sections as described previously,^1^ 15 and 250K Nsp Assay Kits (Affymetrix, Santa Clara, CA) were used according to the manufacturer’s protocol, except for increased starting genomic DNA. One microgram of genomic DNA was digested with Nsp restriction enzyme, ligated to adaptors, and amplified by polymerase chain reaction (PCR) using a universal primer. After purification of PCR products with single nucleotide polymorphism (SNP) Clean magnetic beads (Agencourt Biosciences, Beverly, MA), amplicons were quantified, fragmented, labeled, and hybridized to 250K SNP arrays. After washing and staining, the arrays were scanned to generate CEL files for downstream analysis. Data acquired from the Affymetrix Gene-Chip Operating System v4.0 were analyzed using Affymetrix Gene-Chip Genotyping Analysis Software v4.1. Copy number analysis was performed with Copy Number Analyzer for Affymetrix GeneChip arrays v3.0, as previously described.^1^

### Multiregion Gene Sequencing

To validate somatic mutations and estimate mutant allele frequencies, we conducted deep Sanger sequencing. To further exclude another subtype of renal cell carcinoma, sequencing was performed for mutation discovery of known genes involved in renal cell carcinomas. PCR sequences were chosen for the frequently mutated genes in renal cell carcinoma. These genes included VHL (von Hippel–Lindau tumor suppressor), cMET (MET proto-oncogene [hepatocyte growth factor receptor]), FLCN (folliculin [Birt–Hogg–Dubé protein]), and FH (fumarate hydratase) genes. DNA was obtained from 10 µm paraffin sections from the same tissue blocks used in the SNP-based array analysis. A normal kidney tissue was matched with oncocytoma-like regions and high-grade oncocytic carcinoma.

### Microarray Whole Genome mRNA Expression Analysis

Using an Illumina HumanHT-12 v4.0 Whole-Genome cDNA-mediated Annealing, Selection, Extension, and Ligation (DASL) (WGDASL) platform (Illumina, San Diego, CA), expression data were obtained using 29,378 gene probes representing 18,401 known and predicted genes. Both the benign oncocytoma-like regions and the regions of high-grade oncocytic carcinoma were analyzed with the same tumor tissue blocks and matched normal control used in the SNP-based array analysis. Normalization and background subtraction was performed using Genome Studio Software (Illumina). After data normalization, expression ratios of messenger RNA (mRNA) were calculated over the normal control. Overexpression and underexpression were defined as a 2-fold difference when compared with expression values of normal control kidney tissue. Similar expression was when genes had ratios between 0.8 and 1.2 to the normal control kidney tissue.

## RESULTS

### Morphologic Findings

**FIGURE 1:**
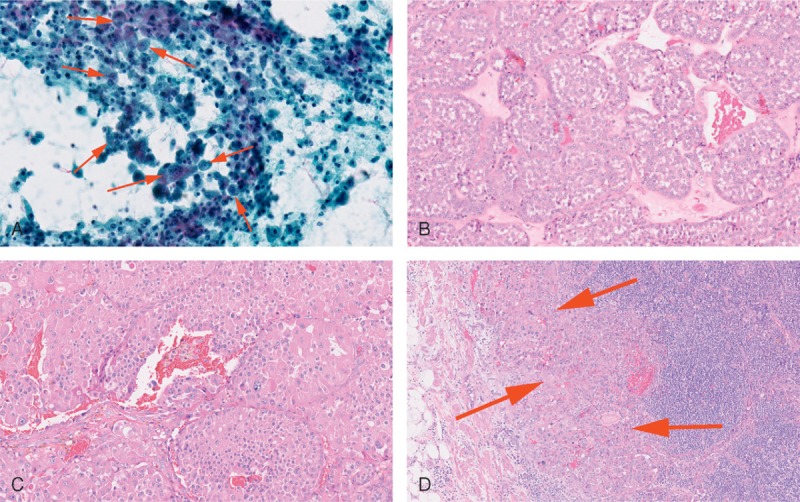
(A) FNA of the aortocaval lymph node (20× objective). (B) Benign oncocytoma-like region of the renal tumor (20× objective). (C) High-grade oncocytic carcinoma region of the renal tumor (20× objective). (D) Aortocaval lymph node with metastatic tumor (10× objective). FNA = fine needle aspirate.

The initial FNA of an aortocaval lymph node showed large oncocytic cells (red arrows) with anaplastic features (Figure 1A, 20× objective). In the resection specimen, approximately one third of the renal tumor histologically showed regions classic for oncocytoma (Figure 1B, 20× objective). There was nested architecture and small round cells with round nuclei. Cytoplasmic clearing or nuclear features suggestive of an eosinophilic chromophobe carcinoma were absent. Two thirds of the renal mass showed sheets of high-grade anaplastic cells, atypical mitoses, hemorrhage, and necrosis (Figure 1C, 20× objective). These regions of high-grade carcinoma merged with the benign oncocytoma-like regions without any sharp demarcation. Immunohistochemistry for CD10, vimentin, and renal cell carcinoma antigen were entirely negative, arguing against a collision between an oncocytoma and a high-grade clear cell renal cell carcinoma and supporting a progression of the disease. The aortocaval lymph node (Figure 1D, 10× objective) showed a metastatic collection of cells (red arrows) morphologically similar to the regions of high-grade oncocytic carcinoma in the renal tumor.

### Genomic Analysis

#### Virtual Karyotype With SNP-Based Array Analysis and Immunohistochemical Support

**FIGURE 2:**
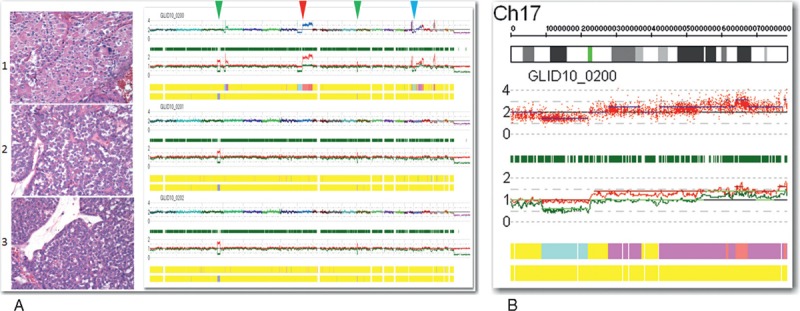
(A) Virtual karyotype using SNP-based array analysis. (B) Magnification of the heterozygous deletion in 17p that houses FLCN at 17p11.2. FLCN = folliculin (Birt–Hogg–Dubé protein).

The benign oncocytoma-like regions (samples 2 and 3 in Figure 2A) showed no significant genomic abnormalities consistent with the morphologic impression of oncocytoma.^1,2^ However, a unique genomic profile was present in the region of high-grade oncocytic carcinoma (sample 1 in Figure 2A). There was a large heterozygous deletion in the region of 17p (blue arrowhead, sample 1 in Figure 2A and region magnification in Figure 2B). The deleted 17p locus included FLCN (at 17p11.2), the gene mutated in Birt–Hogg–Dubé Syndrome (BHDS). There was also a loss of 8p and gain of 8q (red arrowhead, sample 1 in Figure 2A) in the high-grade oncocytic carcinoma. Losses of 8p have been reported in some cases of locally advanced clear cell renal cell carcinoma and metastases.^3,4^ Gains of 8q in renal cell carcinoma have been associated with metastases and poor survival.^5^ In addition, all 3 samples showed 2 incidental regions of autozygosity on 3q and 12p (green arrowheads, samples 1, 2, and 3 in Figure 2A) for which the significance is unknown.

**FIGURE 3:**
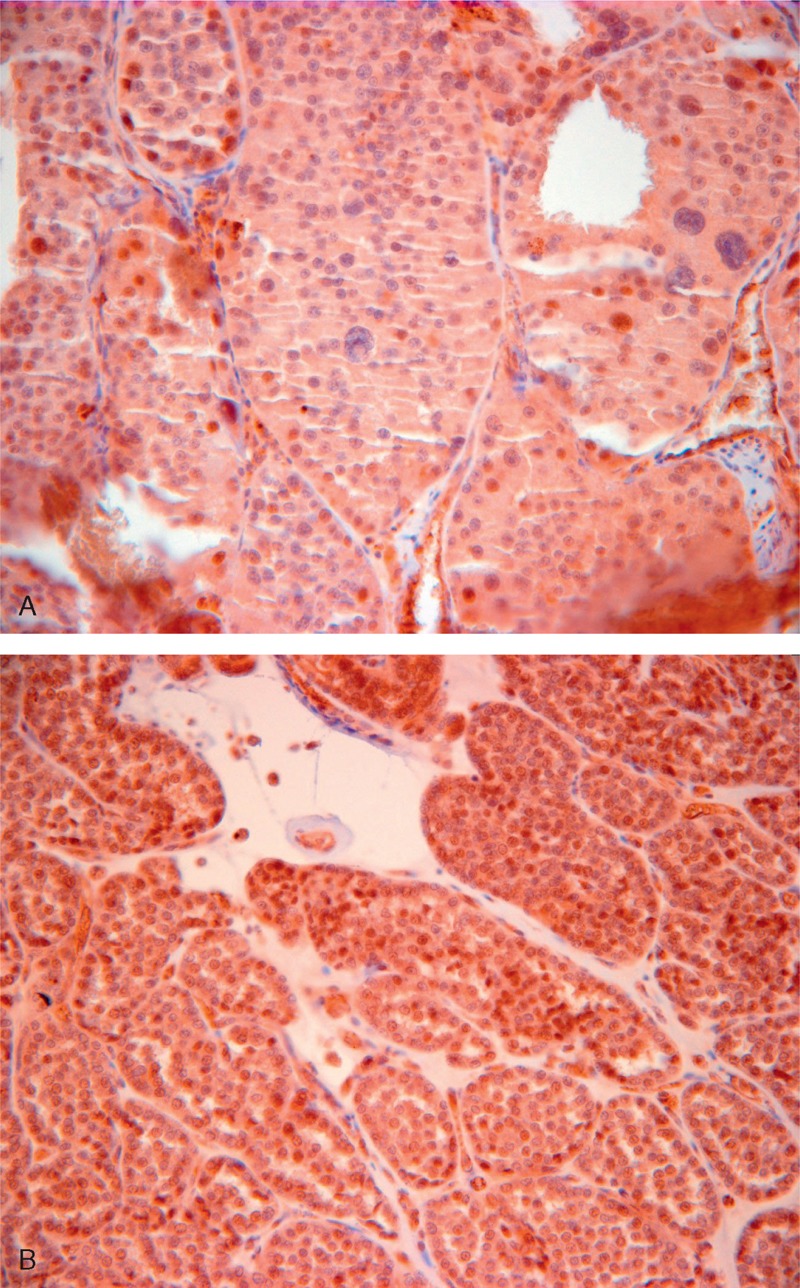
(A) and (B) Immunohistochemical FLCN protein expression. (A) High-grade oncocytic carcinoma. (B) Benign oncocytoma-like region with slightly higher protein expression of FLCN. FLCN = folliculin (Birt–Hogg–Dubé protein).

Protein expression of FLCN was explored through immunohistochemistry (Figure 3A and B). The high-grade oncocytic carcinoma region showed decreased protein expression of FLCN (Figure 3A) when compared with the benign oncocytoma-like regions (Figure 3B). This decrease in protein expression appeared to correspond to the heterozygous deletion seen at 17p11.2 on virtual karyotype.

### Multiregion Gene Sequencing

The overall findings of multiregion gene sequencing were provided in Table 1. No variant was detected in the VHL, cMET, or FH genes. This supported exclusion of other subtypes of renal cell carcinoma such as clear cell carcinoma, papillary carcinoma, and rare cases of renal cell carcinoma associated with hereditary leiomyomatosis and renal cell carcinoma. With FLCN, known intronic SNP variants were present in introns 5 and 8. In addition, we detected a known silent polymorphism in exon 11. Neither the high-grade oncocytic carcinoma region nor the oncocytoma-like regions contained any clinically significant mutations.

**TABLE 1:**
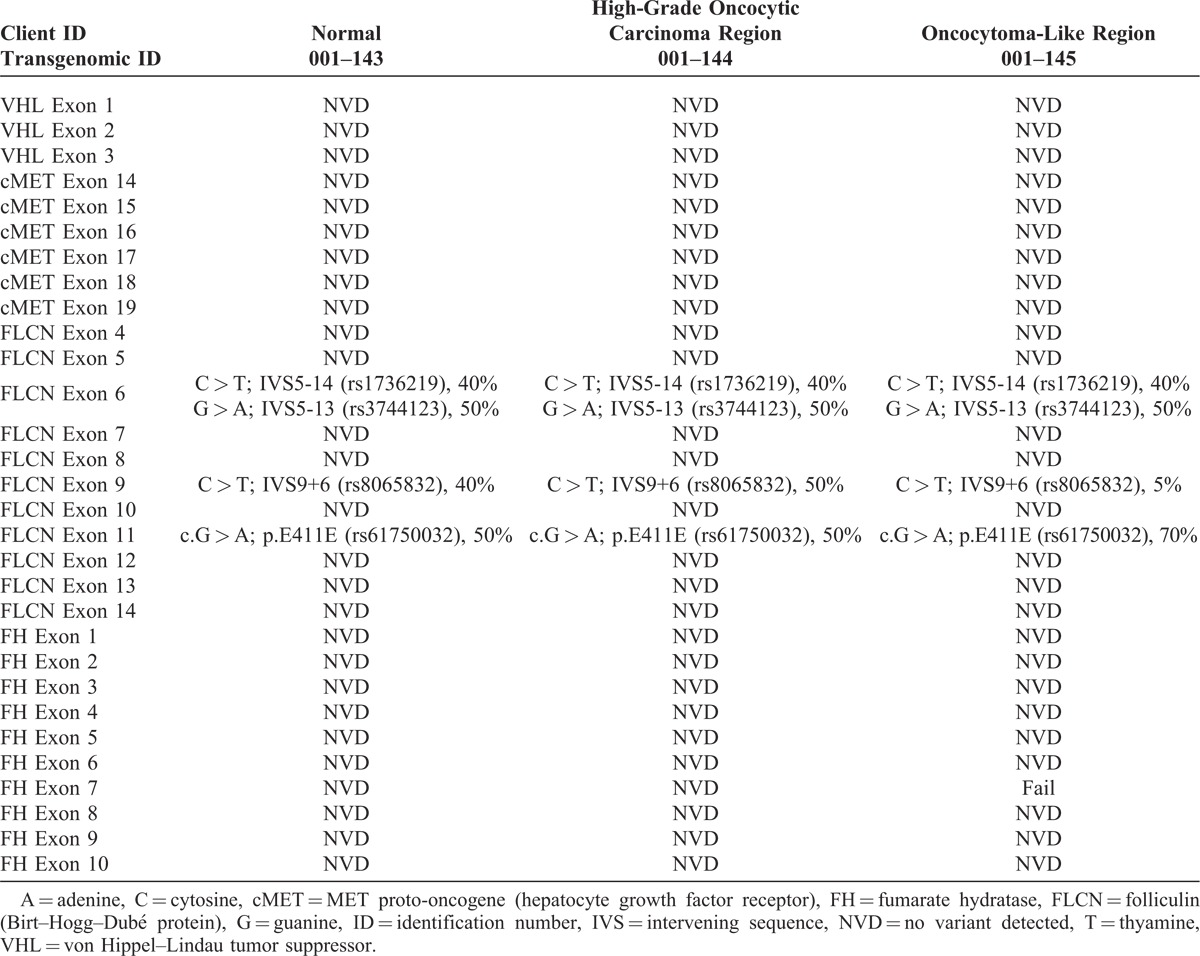
Multiregion Gene Sequencing for Known Genes Involved With Renal Cell Carcinoma

### Microarray Whole Genome mRNA Expression Analysis

**FIGURE 4:**
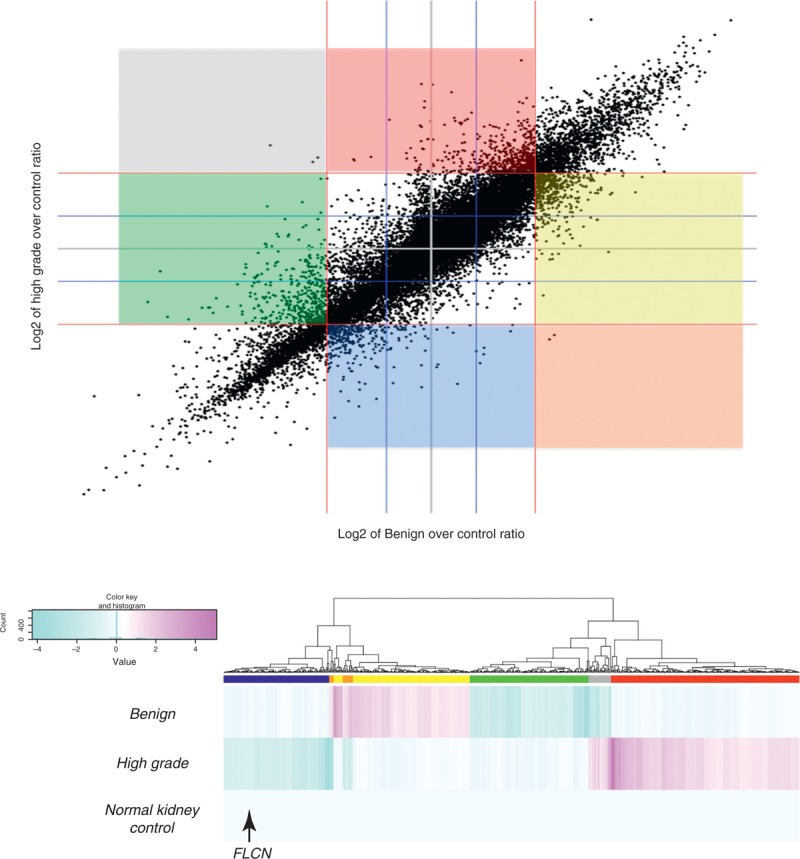
(A) Scatter plot of expression data for 29,378 probes. The colored sections represent the following: blue = high-grade downregulated, red = high-grade upregulated, green = benign downregulated, yellow = benign upregulated, grey = high-grade upregulated and benign downregulated, orange = high-grade downregulated and benign upregulated. (B) Hierarchical clustering and heat map of expression ratios for 748 transcripts relative to the normal kidney. The genes falling within the 6 colored sectors in (A) and the gene probes corresponding to the 6 colored bars beneath the dendrogram in (B) represent genes that showed 2-fold overexpression or underexpression in 1 region with the other region having the opposite 2-fold expression or similar expression relative to normal kidney. FLCN = folliculin (Birt–Hogg–Dubé protein).

Using an Illumina HumanHT-12 v4.0 WGDASL platform, a scatter plot was generated in Figure 4A showing an overview of the expression ratios over the normal control. Supplementary Table S1 (http://links.lww.com/MD/A35) provides the raw data. Overall, expression ratios of both tumor regions were concordant further supporting a common lineage to the tumor. Genes showing a 2-fold overexpression in both the benign oncocytoma-like regions and high-grade oncocytic carcinoma regions included several notable genes such as VHL, HIF1A (hypoxia inducible factor 1), cMET, and TSC1 (tuberous sclerosis 1), which are cancer genes involved in renal cell carcinoma (Table 2). Other notable genes include BCL2 (B-cell CLL/lymphoma 2), MYC (v-myc avian myelocytomatosis viral oncogene homolog), BRCA1 (breast cancer 1, early onset), and MDM2 (MDM2 proto-oncogene), which are involved in cell death, survival, DNA repair, growth, and proliferation. Commonly overexpressed genes potentially serving as biomarkers for targeted therapy include EGFR (epidermal growth factor receptor), KIT (v-kit Hardy-Zuckerman 4 feline sarcoma viral oncogene homolog), PDGFRL (platelet-derived growth factor receptor-like), VEGFA (vascular endothelial growth factor A), and VEGFB (vascular endothelial growth factor B). The KIT gene showed the highest overexpression.

**TABLE 2:**
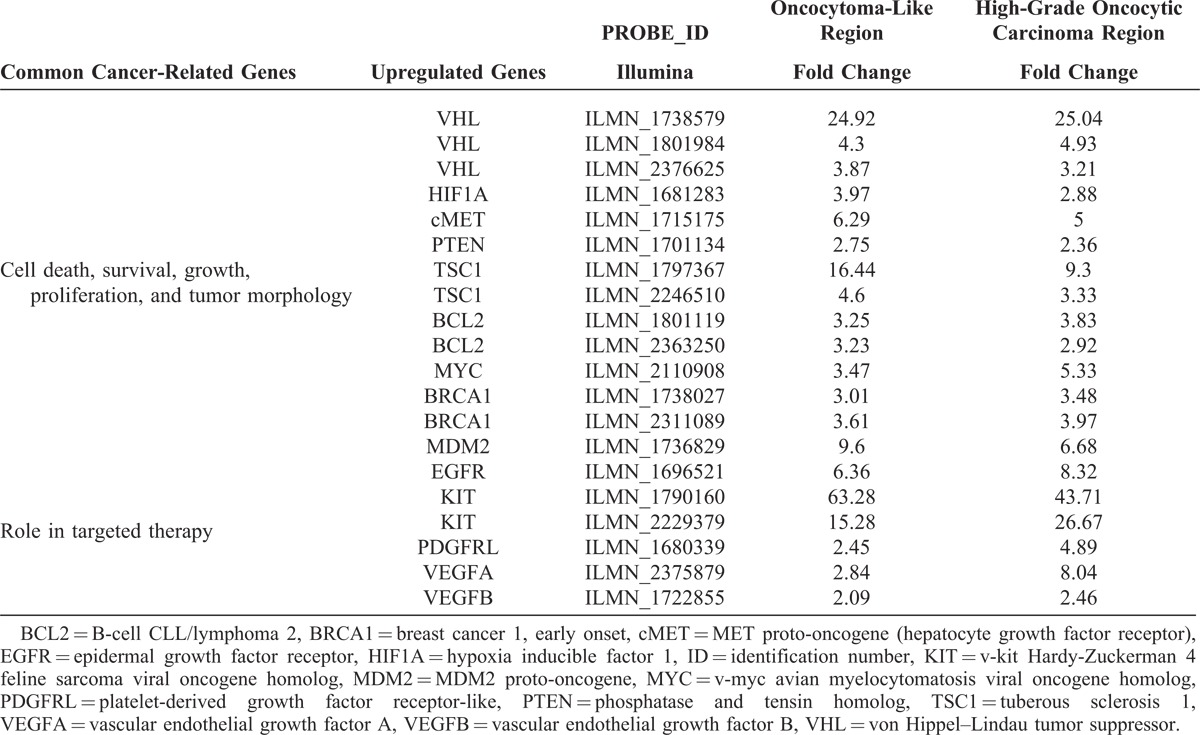
Cancer-Related Genes Involved With Renal Cell Carcinoma, Cell Death, Survival, Growth, Proliferation, Tumor Morphology, and Targeted Therapy

Despite the overall similarity of gene expression between the 2 tumoral regions, differences were also exhibited. Figure 4B and Supplementary Table S2 (http://links.lww.com/MD/A36) show 6 distinct gene subsets: underexpressed in the high-grade oncocytic carcinoma region but no change in the benign oncocytoma-like regions (137 gene probes; note that FLCN falls within this section of the dendrogram, which is consistent with previous SNP array and immunohistochemical results); underexpressed in high-grade oncocytic carcinoma region and overexpressed in the benign oncocytoma-like regions (19 gene probes); no change in the high-grade oncocytic carcinoma region but overexpressed in the benign oncocytoma-like regions (164 gene probes); no change in the high-grade oncocytic carcinoma region but underexpressed in the benign oncocytoma-like regions (154 gene probes); overexpressed in the high-grade oncocytic carcinoma region and underexpressed in the benign oncocytoma-like regions (29 gene probes); overexpressed in the high-grade oncocytic carcinoma region but no change in the benign oncocytoma-like regions (245 gene probes). Supplementary Table S3 (http://links.lww.com/MD/A37) shows a filtered gene probe list derived from subsets 1 and 6 in Supplementary Table S2 (http://links.lww.com/MD/A36), which reflect differences in expression in the high-grade oncocytic carcinoma region but no change in the benign oncocytoma-like regions. The 8 underexpressed gene probes in subset 1 and 19 overexpressed gene probes in subset 6, corroborates the 8p loss and 8q gain seen on virtual karyotype of the high-grade oncocytic carcinoma region.

## DISCUSSION

The existence of a malignant oncocytoma has been debated due to general acceptance of renal oncocytoma as benign. Rare cases of metastatic oncocytoma have been reported, but these cases may have represented clear cell carcinoma and/or chromophobe carcinoma.^6,7^ A progression from renal oncocytoma to oncocytic carcinoma has not yet been reported. The oncocytic carcinoma region in this tumor showed overwhelming sheet-like growth, desmoplastic stroma, necrosis, marked nondegenerative atypia, and frequent atypical mitoses. Perez-Ordonez et al^7^ evaluated 70 oncocytomas and reported no atypical mitoses. Morell-Quadreny et al^8^, described 6 “atypical oncocytomas” that came closest in appearance to our tumor. These tumors had cellular pleomorphism, transcapsular invasion, and focal necrosis but did not show the confluent necrosis and atypia in our case. A cytogenetic evaluation was performed on these “atypical oncocytomas” with none of the similar abnormalities of 17p, 8p, or 8q seen in our tumor.^8^

A diagnostic consideration is chromophobe renal cell carcinoma (eosinophilic type), however, the multiple aberrations of chromophobe renal cell carcinoma with loss of chromosomes^1,2^ 1, 2, 6, 10, 13, and 17 were not present in any of the regions of our tumor. Another consideration is the “hybrid oncocytic/chromophobe” kidney tumor (HOT), as described by Petersson et al.^9^ Such tumors show diffuse hybrid morphology between oncocytoma and chromophobe with multiple numeric chromosomal aberrations. Our tumor lacked diffuse morphologic changes and rather showed a dichotomous histology with benign oncocytoma-like regions admixed with high-grade oncocytic carcinoma regions. HOT also generally behaves in an indolent manner.^9^

The virtual karyotype using SNP-based array analysis allowed us to focus on genomic aberrations found in the regions of high-grade oncocytic carcinoma, notably the 17p region, which implicated the FLCN gene, along with 8p and 8q where there was a loss and gain, respectively. Through sequencing of genes known to be associated with renal carcinomas, we were able to further argue against a collision tumor with other histologic subtypes. The whole genome microarray expression analysis enabled us to support a common lineage to the benign oncocytoma-like regions and regions of high-grade oncocytic carcinoma.

The heterozygous deletion of the 17p region with FLCN underexpression appears somatic. This contrasts other renal oncocytic tumors “hybrid chromophobe-oncocytomas” of BHDS, which is caused by germline FLCN mutations.^10^ BHDS is an autosomal dominant hereditary cancer syndrome caused by germline mutations in FLCN gene. Clinically, it presents with small skin tumors (fibrofolliculomas), pulmonary cysts and renal tumors of which oncocytic and chromophobe types predominate. Evidence for a somatic aberration in our patient are several-fold: the patient lacked a family history suspicious for BHDS and had no FLCN mutation in any tissue analyzed, including normal kidney; only the regions of high-grade oncocytic carcinoma showed the heterozygous deletion of the region housing FLCN on SNP array; FLCN was underexpressed only in the high-grade oncocytic carcinoma region both at the protein and transcript level.

The heterozygous deletion of the 17p region with FLCN underexpression is but 1 factor contributing to the aggressive pathobiology of the high-grade oncocytic carcinoma region. Mechanistically, the underexpression of FLCN complex in the region of high-grade oncocytic carcinoma would dysregulate ERK (extracellular-signal-regulated kinase)/mTOR (mechanistic target of rapamycin [serine/threonine kinase]) and AMP-activated protein kinase complexes and lead to apoptotic resistance through an aberrant transforming growth factor-β mediated transcription/signaling.^11–14^ FLCN underexpression facilitates invasion through increased MMP9 (matrix metallopeptidase 9) expression.^11,12^ However, the aggressive phenotype demonstrated by this tumor is beyond other low-grade FLCN-mediated renal oncocytic tumors such as HOT “hybrid chromophobe-oncocytomas” of BHDS.^8^ This, in turn, hints at additional contributory mechanisms.

FLCN underexpression increases MMP9 expression which is further potentiated by HDAC4 (histone deacetylase 4) underexpression.^15,16^ Overexpression of an additional metalloproteinase, matrix metallopeptidase 12, further potentiated the invasiveness of high-grade oncocytic carcinoma region. Furthermore, notable overexpressed genes like SRC (SRC proto-oncogene) in the high-grade oncocytic carcinoma region can drive transformation,^17^ proliferation, growth,^18^ and invasion.^19^ Using the “Knowledge Base” of Ingenuity Pathway Analysis library (Ingenuity Systems, www.ingenuity.com), the overexpression of genes on 8q (Supplementary Table 3, http://links.lww.com/MD/A37) was associated with various cancer types and cell lines with some having roles in invasiveness, proliferation, transformation, progression, PI3K (phosphoinositide 3-kinase), Akt (v-akt murine thymoma viral oncogene homolog), ERK, and nuclear factor of κ light polypeptide gene enhancer in B-cells pathway activation. As such, our global transcriptomic analysis revealed an even more aggressive phenotype for the high-grade oncocytic carcinoma region than that of HOT “hybrid chromophobe-oncocytomas” of BHDS mediated by FLCN.

Intriguing gene expression patterns present in the benign oncocytoma-like regions were the underexpression of metalloproteinases matrix metallopeptidase 1 and matrix metallopeptidase 7 diminishing invasiveness. Claudin 8, paired box 8,^20,21^ and KIT overexpression in the oncocytoma-like regions further corroborated the tumor as being from a renal oncocytoma-like lineage. Interestingly, several cancer-related genes (VHL, HIF1A, cMET, phosphatase and tensin homolog, TSC1, BCL2, BRCA1), which include some tumor suppressors, were also found to be overexpressed in both the benign oncocytoma-like region and the high-grade oncocytic carcinoma region. Although overexpression of some of these genes was predictable for the high-grade oncocytic carcinoma region, overexpression was a puzzling observation for the benign oncocytoma-like regions. There was no database to refer if overexpression of these genes is a consistent finding in renal oncocytoma. It is possible that overexpression of these cancer-related genes could be used to discriminate for malignant potential in renal oncocytoma-like tumors with borderline morphologic features.

Our patient’s disease was controlled for close to 3 years while being on tyrosine kinase inhibitors. This was despite a metastatic clinical presentation. The overexpression of EGFR, KIT, PDGFRL, VEGFA, VEGFB, would predict for therapeutic effectiveness for these tyrosine kinase inhibitors. With VEGFA, expression was increased >5-fold in the high-grade oncocytic carcinoma region, possibly facilitating angiogenesis further. This may be attributed to several genes (HDAC4,^22^ SRC,^23^ and mothers against DPP homolog^24^ family member 7), the expression patterns of which would account for upregulation of VEGF. Sunitinib and Axitinib are the 2 drugs that may have the most effect as KIT is one of the overexpressed genes. Because there appears to be a dysregulation/activation of PI3K, Akt, MAPK (mitogen-activated protein kinase)/ERK, and mTOR pathways in the high-grade oncocytic carcinoma region, the addition of an mTOR inhibitor such as Temsirolimus would provide an additive effect for his targeted therapy. Interestingly, MYC is overexpressed in both the benign oncocytoma-like and high-grade oncocytic carcinoma regions. MYC located on 8q was almost 2-fold higher in the region of high-grade oncocytic carcinoma, and this may have reflected the 8q gain seen on SNP array. MYC overexpression would also predict a therapeutic benefit with specific c-Myc inhibitors or agents that target the MAPK/ERK pathway.^5^

In conclusion, this was the first reported case of a benign oncocytoma transforming to high-grade oncocytic carcinoma, supported by molecular genetic profiling of different tumoral regions that highlighted the importance of tumor heterogeneity in all aspects of patient management. This case report also re-emphasizes the need for proper sampling and analysis of (histologically) heterogeneous tumor regions. Identification of potentially druggable molecular alterations in different regions and sites may improve patient management and outcomes.^25,26^
